# Strong Interfacial Perpendicular Magnetic Anisotropy in Exchange-Biased NiO/Co/Au and NiO/Co/NiO Layered Systems

**DOI:** 10.3390/ma14051237

**Published:** 2021-03-05

**Authors:** Mateusz Kowacz, Błażej Anastaziak, Marek Schmidt, Feliks Stobiecki, Piotr Kuświk

**Affiliations:** 1 Institute of Molecular Physics, Polish Academy of Sciences, Mariana Smoluchowskiego 17, 60-179 Poznań, Poland; mateusz.kowacz@ifmpan.poznan.pl (M.K.); blazej.anastaziak@ifmpan.poznan.pl (B.A.); schmidt@ifmpan.poznan.pl (M.S.); feliks.stobiecki@ifmpan.poznan.pl (F.S.); 2 NanoBioMedical Centre, Adam Mickiewicz University in Poznan, Wszechnicy Piastowskiej 3, 61-614 Poznań, Poland

**Keywords:** perpendicular magnetic anisotropy, exchange bias, magnetic thin films, antiferromagnetic oxides

## Abstract

The ability to induce and control the perpendicular magnetic anisotropy (PMA) of ferromagnetic layers has been widely investigated, especially those that offer additional functionalities (e.g., skyrmion stabilization, voltage-based magnetization switching, rapid propagation of domain walls). Out-of-plane magnetized ferromagnetic layers in direct contact with an oxide belong to this class. Nowadays, investigation of this type of system includes antiferromagnetic oxides (AFOs) because of their potential for new approaches to applied spintronics that exploit the exchange bias (EB) coupling between the ferromagnetic and the AFO layer. Here, we investigate PMA and EB effect in NiO/Co/Au and NiO/Co/NiO layered systems. We show that the coercive and EB fields increase significantly when the Co layer is coupled with two NiO layers, instead of one. Surrounding the Co layer only with NiO layers induces a strong PMA resulting in an out-of-plane magnetized system can be obtained without a heavy metal/ferromagnetic interface. The PMA arises from a significant surface contribution (0.74 mJ/m^2^) that can be enhanced up to 0.99 mJ/m^2^ by annealing at moderate temperatures (~450 K). Using field cooling processes for both systems, we demonstrate a wide-ranging control of the exchange bias field without perturbing other magnetic properties of importance.

## 1. Introduction

For many years, magnetic thin films have aroused great interest related to their potential uses in information technology and spintronics. For such applications, among many different properties, the most important are those that determine the magnetization reversal process and the stability of the magnetic configuration at remanence. Multilayer systems composed of ferromagnetic (FM) layers surrounded by non-ferromagnetic layers, usually heavy metals (HM) (e.g., Au, Pt, Pd [1,2,3]), exhibit surface anisotropy of the Néel type [4] that enables strong perpendicular magnetic anisotropy (PMA) and greatly stabilizes the magnetic configurations of these nanostructures [5]. Apart from PMA, the interactions between the FM layers and the surrounding layers are crucial modifiers of the magnetization reversal process. In particular, the exchange bias (EB) coupling occurring at the FM/antiferromagnetic (AF) interface [6] causes unidirectional anisotropy. The presence of this type of anisotropy is manifested in the asymmetry of the magnetization reversal process with respect to reversals of the magnetic field [7,8].

In addition to magnetic properties, the electrical properties of multilayers are also decisive in spintronic applications. Therefore, many studies have focused on layered systems consisting of FM layers surrounded by non-conductive metal oxide layers (MO) [9,10,11,12,13]. Current extensive research on such system reveals that, at the FM/MO interfaces, the MO layers induce strong PMA [9,14,15] and interfacial Dzyaloshinskii-Moriya interaction [16,17]. These responses can also be triggered using antiferromagnetic oxides (AFOs) [18,19,20], enabling new ways to tailor magnetization reversal through the EB coupling. This capability can be used to stabilize skyrmions at room temperature (RT) without external magnetic fields [21,22,23]. The EB effect induced by coupling FM with AFO might be particularly important to domain wall (DW) pinning [24], which is essential for the stabilization and optimization of the DW movement in racetrack memories [25,26].

To date, research has mainly focused on HM/FM/AFM systems, where the heavy metal (HM) induces interfacial contribution to PMA in the FM layer, and the single antiferromagnetic (AFM) layer provides EB coupling. This means that the key properties of these systems are separately activated at different interfaces, making strong interfacial PMA and large EB field ($H_{\mathrm{EB}}$) difficult to obtain simultaneously. However, because EB coupling originating from both interfaces may show additive behavior [27], a large $H_{\mathrm{EB}}$ should be achievable by coupling the FM to AFMs on both sides. Therefore, it is essential to find suitable AFM materials to surround the FM layer so that both AFM/FM and FM/AFM interfaces simultaneously support strong PMA and high $H_{\mathrm{EB}}$. These systems should also offer the ability to tune $H_{\mathrm{EB}}$ and coercive field ($H_{C})$, which is of particular interest to design layered stacks suitable for applications [28]. A good candidate is Co/NiO, because NiO favors both of these effects at RT [29]. Moreover, NiO is an insulator with good magnetotransport properties, useful as a barrier in magnetic tunnel junctions [30,31] or as a conductor of spin current [32,33,34] in oxide-based spintronic devices. The electrical insulating properties of NiO are also used to control the EB effect by the electric field [35], which opens a way to realize multifunctional devices with low power consumption. Furthermore, the development of layered systems in which the FM layer is surrounded only by a dielectric layer may improve the efficiency of spin-transfer torque-driven domain wall motion due to an increase in the current density flowing in the FM layer.

In this paper, we investigate EB and PMA in NiO^b^/Co/Au, and in a double-exchange biased NiO^b^/Co/NiO^t^ systems (the superscript b and t denote the bottom and top NiO layer, respectively) that has not been studied so far. We show that in these systems, the AFM–FM interface supports a strong PMA caused by surface contributions to the effective anisotropy, with similar values as in HM/FM/HM systems. Moreover, because EB coupling between Co and both antiferromagnetic NiO layers is an additive effect, $H_{\mathrm{EB}}$ reaches a large value of 45 mT. Additionally, we showed that a field cooling (FC) process enhances the PMA, which in turn allows for tuning *H*_EB_ in a wide range.

## 2. Experiment

**Figure 1:**
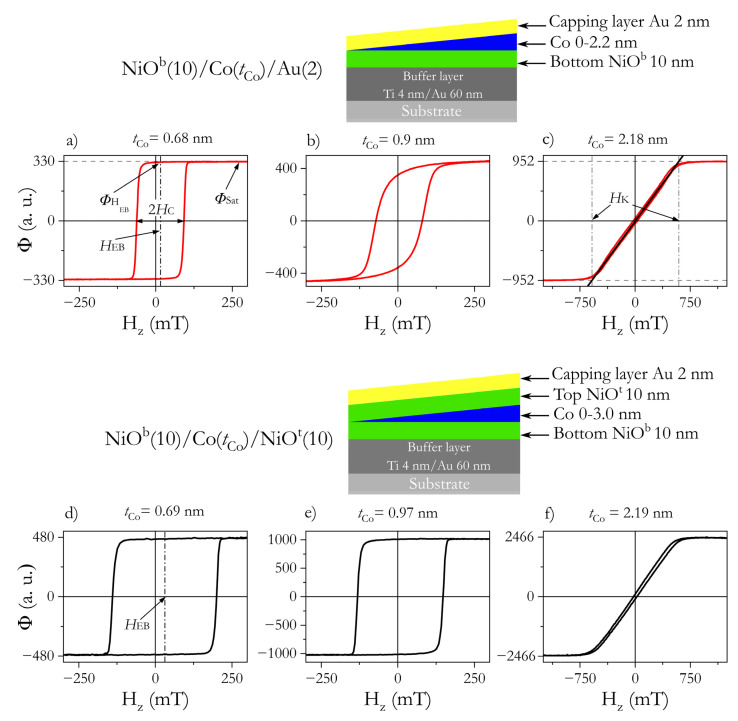
Morphology of samples and representative PMOKE hysteresis loops for the NiO^b^/Co-wedge/Au system: (**a**) $t_{\mathrm{Co}}$ = 0.68 nm, (**b**) $t_{\mathrm{Co}}$ = 0.9 nm, (**c**) $t_{\mathrm{Co}}$ = 2.18 nm and for the NiO^b^/Co-wedge/NiO^t^ system: (**d**) $t_{\mathrm{Co}}$ = 0.69 nm, (**e**) $t_{\mathrm{Co}}$ = 0.97 nm, (**f**) $t_{\mathrm{Co}}$ = 2.19 nm.

This work describes two systems: NiO^b^(10 nm)/Co(wedge shape: 0–2.2 nm)/Au(2 nm) and NiO^b^(10 nm)/Co(wedge shape: 0–3 nm)/NiO^t^(10 nm)/Au(2 nm) deposited on naturally oxidized silicon substrates with Ti(4 nm)/Au(60 nm) buffers (Figure 1). The Co thickness gradient is 0.15 and 0.27 nm/mm for the NiO^b^/Co/Au and NiO^b^/Co/NiO^t^ systems, respectively. The thicknesses of the layers were calibrated using X-ray reflectivity and a quartz balance. The wedge-shaped Co layer was deposited using a shutter movement with constant velocity calculated according to the deposition rate. The samples were fabricated in a PREVAC (Rogów, Poland) ultra-high vacuum (UHV) system with three chambers for distinct deposition technologies: magnetron sputtering (MS), pulsed laser deposition (PLD), and ion beam sputtering. The Ti, Au, and Co layers were deposited using MS in an argon-rich atmosphere ($p_{\mathrm{Ar}}$ = 1 × 10^−4^ mbar), and the NiO layer was deposited by PLD in an oxygen-rich atmosphere ($p_{O}$ = 1.5 × 10^−5^ mbar) [36]. For deposition, we used an ultra-pure Ti (Testbourne Ltd., Basingstoke, UK), Au (Mennica Metale Szlachetne S.A., Warsaw, Poland), Co (Kurt J. Lesker Company Ltd., Hastings, UK), and stoichiometric NiO (MaTeck GmbH, Jülich, Germany) targets. The transfer between MS and PLD chambers is done through a distribution chamber without breaking UHV conditions (during transfer, p ≤ 5 × 10^−8^ mbar). Nevertheless, formation of an ultrathin CoO layer at the Co/NiO^t^ interface in NiO^b^/Co/NiO^t^ structure is expected during deposition of NiO in the oxygen-rich atmosphere [36,37]. To stabilize the $H_{\mathrm{EB}}$ in an as-deposited state, all depositions took place in perpendicular external magnetic fields ($H_{\mathrm{dep}}$ = −185 mT).

The magnetic properties of the NiO^b^/Co/Au and the NiO^b^/Co/NiO^t^ systems were measured at RT along the Co thickness gradient using a polar magneto-optical Kerr effect (PMOKE) magnetometer. The measurements were performed in two different ranges of external perpendicular magnetic fields ($H_{z}$): (a) between −600 and 600 mT to determine $H_{C}$ and $H_{\mathrm{EB}}$ fields, and (b) between −1500 and 1500 mT to obtain anisotropy fields ($H_{K}$) above the Co thickness ($t_{\mathrm{SRT}}$) at which spin reorientation transition (SRT) from PMA to easy-plane anisotropy (EPA) occurs.

The surface topography of the NiO^b^/Co/Au and the NiO^b^/Co/NiO^t^ samples was measured using atomic force microscopy (Agilent 5500, Santa Clara, CA, USA) in tapping mode. The measurements were performed using an All-In-One atomic force microscope probe (Budget Sensor, Sofia, Bulgaria).

The sign and value of $H_{\mathrm{EB}}$ of the NiO^b^/Co/Au and NiO^b^/Co/NiO^t^ systems were tuned with the following four FC steps. Each step took place in a vacuum chamber (p = 1 × 10^−6^ mbar), starting from RT to a given temperature ($T_{\mathrm{FC}}$) with a heating rate ~16 K/min. After 5 min isothermal annealing at $T_{\mathrm{FC}}$, the sample was cooled down to RT with a fixed orientation of perpendicular magnetic field ($H_{\mathrm{FC}}$ = ±170 mT) (Table 1).

## 3. Results and Discussion

### 3.1. Magnetic Properties of NiO^b^/Co/Au and NiO^b^/Co/NiO^t^ Systems in As-Deposited State

Figure 1 shows three representative PMOKE hysteresis loops for both investigated systems measured at different $t_{\mathrm{Co}}.$ For thin Co layers, the rectangular shape of hysteresis loops with $\phi_{H_{\mathrm{EB}}}$/$\phi_{\mathrm{Sat}}$ = 1 ($\phi_{H_{\mathrm{EB}}}$ and $\phi_{\mathrm{Sat}}$ are PMOKE signals at $H_{\mathrm{EB}}$ and saturation, respectively) (Figure 1a,d,e) shows that both systems exhibit PMA. In the case of NiO^b^/Co/Au with $t_{\mathrm{Co}}$ slightly below SRT, the loop shape suggests (Figure 1b) that a small in-plane magnetization component exists at remanence. In all cases, a positive $H_{\mathrm{EB}}$ (the hysteresis loop shift from $H_{z}$ = 0 is opposite to $H_{\mathrm{dep}}$) is also clearly visible, indicating that the EB coupling is parallel to $H_{\mathrm{dep}}$. For thicker Co, the system undergoes SRT and exhibits EPA (Figure 1c,f).

Before comparing results for the entire $t_{\mathrm{Co}}$ range, it should be emphasized that the dependence of the PMOKE signals versus $t_{\mathrm{Co}}$ (*ϕ*($t_{\mathrm{Co}}$)) of NiO^b^/Co/NiO^t^ are shifted by about Δ$t_{\mathrm{Co}}$ = 0.26 nm with respect to NiO^b^/Co/Au and Au/Co/Au systems [36]. We have previously shown that the deposition of the NiO layer in an oxygen-rich atmosphere results in the formation of a thin CoO layer between Co and NiO at the Co/NiO^t^ interface [36,37]. The samples in this report were deposited in the same conditions; therefore, a similar CoO layer should form in NiO^b^/Co/NiO^t^. Comparisons of the present data with the NiO^b^/Co/Au and Au/Co/Au systems should be based on the real Co thickness (without CoO); therefore, the data in this paper were adjusted based on Δ$t_{\mathrm{Co}}$ determined above.

**Figure 2:**
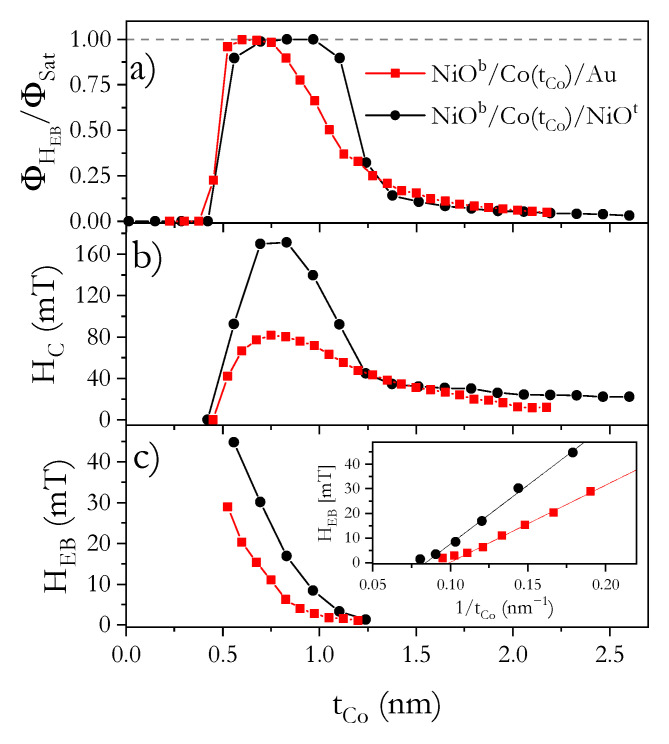
Dependence of normalized Kerr signal ($\phi_{H_{\mathrm{EB}}}$/$\phi_{\mathrm{Sat}}$) at *H* = $H_{\mathrm{EB}}$ (**a**), coercivity ($H_{C}$) (**b**) and exchange bias fields ($H_{\mathrm{EB}}$) (**c**) on Co layer thickness ($t_{\mathrm{Co}}$) for NiO^b^/Co/Au and NiO^b^/Co/NiO^t^ systems. Inset in (c) shows $H_{\mathrm{EB}}$ (1/$t_{\mathrm{Co}}$) dependence.

**Figure 3:**
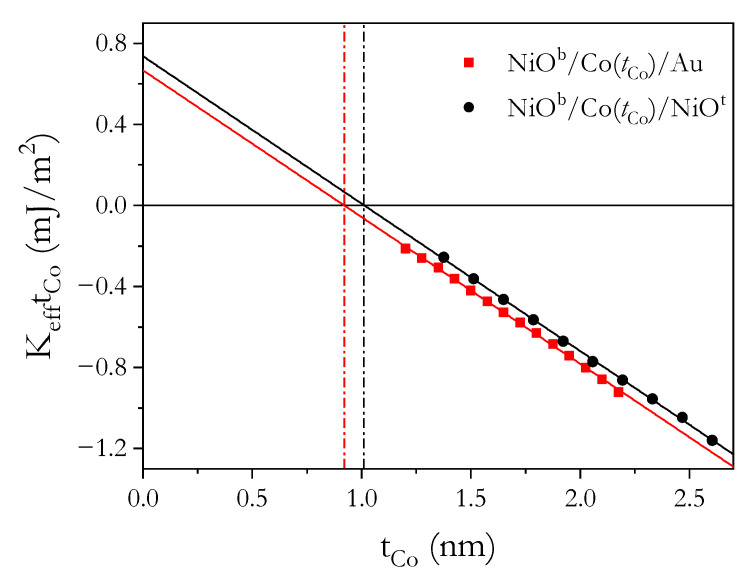
$K_{\mathrm{eff}}t_{\mathrm{Co}}$ vs. $t_{\mathrm{Co}}$ for NiO^b^/Co/Au and NiO^b^/Co/NiO^t^ systems in the as-deposited state. The dashed lines indicate Co thickness corresponding to spin reorientation transition ($t_{\mathrm{SRT}}$ = −2$K_{S}$ /$K_{V}$).

From the analysis of hysteresis loops for NiO^b^/Co/Au, we distinguished three important thickness ranges: (I) for 0.5 nm < $t_{\mathrm{Co}}$ ≤ 0.75 nm, the hysteresis loops are rectangular with sharp corners and $\phi_{H_{\mathrm{EB}}}$/$\phi_{\mathrm{Sat}}$ ≈ 1 (Figure 2a). This is typical of systems with strong PMA in which magnetization reversal takes place by domain nucleation followed by rapid propagation of domain walls [38,39]; (II) for 0.75 nm < $t_{\mathrm{Co}}$ < 0.93 nm, the shape of the hysteresis loops ($\phi_{H_{\mathrm{EB}}}$/$\phi_{\mathrm{Sat}}$ < 1, Figure 1b) indicates that the activation of multiple nucleation centers defines the reversal process [39]. This also suggests that there is a small in-plane magnetization component at remanence; (III) at $t_{\mathrm{Co}}$ = 0.93 nm, the system undergoes SRT (see Figure 3); and as $t_{\mathrm{Co}}$_,_ grows further, the magnetization reversal process approaches that of coherent magnetization rotations for EPA (Figure 1c).

A similar result was obtained for NiO^b^/Co/NiO^t^, with SRT happening at a slightly thicker Co layer ($t_{\mathrm{Co}}$ = 1 nm) (see Figure 3). For this system, the transition from PMA to EPA appears more abruptly for NiO^b^/Co/Au, where it extends over a much greater $t_{\mathrm{Co}}$ range (Figure 2a). This means that the squareness of the hysteresis loops improves (Figure 1e) when the Co layer is coupled with NiO on both sides (like in ref. [27]). A rectangular loop with sharp corners occurs when the nucleation energy significantly exceeds the energy of DW propagation, and the nucleation energies do not show a significant distribution of values in the sample plane [38]. As the difference between these energies decreases and the dispersion of the nucleation energy increases, the magnetization reversal process will evolve from a situation where it occurs through the creation of a few domains and rapid propagation of DW to a situation where nucleation processes take place in many places at different values of the magnetic field. Since the effect on Co out-of-plane anisotropy is stronger in the Co/NiO^t^ than in the Co/Au interface (this will be discussed later), we attribute the distinct magnetization reversal close to SRT to a higher magnetic anisotropy of the NiO^b^/Co/NiO^t^ system. We should also emphasize that the magnetization reversal process close to SRT can also be influenced by the relation between second- and first-order magnetic anisotropy [40]. Since, for the HM/Co/Oxide systems, the second-order magnetic anisotropy can be large [41,42], this anisotropy might be a source of wider $t_{\mathrm{Co}}$ transition range from PMA to EPA for NiO^b^/Co/Au than for NiO^b^/Co/NiO^t^. An understanding of the role of second-order magnetic anisotropy in both systems needs further investigation.

**Figure 4:**
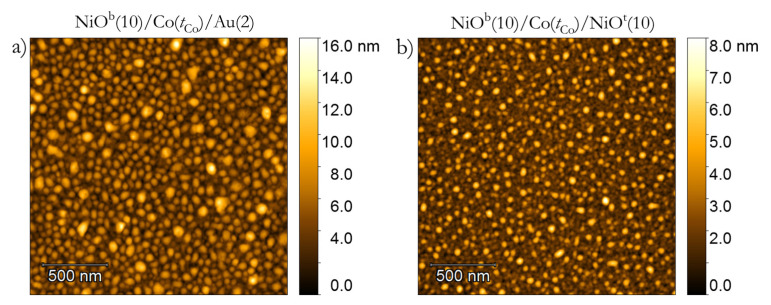
Surface topography measured by atomic force microscope of the as-deposited (**a**) NiO^b^/Co-wedge/Au system at $t_{\mathrm{Co}}$ = 1 nm and (**b**) the NiO^b^/Co-wedge/NiO^t^ system at $t_{\mathrm{Co}}$ = 1 nm.

For both systems (which show polycrystalline structure (Figure 4) with grain sizes below 52 nm for NiO^b^/Co/Au and below 32 nm for NiO^b^/Co/NiO^t^), we found the typical behavior of the EB effect; that is, $H_{\mathrm{EB}}$ is inversely proportional to $t_{\mathrm{Co}}$ (Figure 2c). This reveals that the strong EB coupling is present on both Co/NiO^t^ and NiO^b^/Co interfaces. The most significant difference between our systems in the as-deposited state is related to the values of $H_{C}$ and $H_{\mathrm{EB}}$ (Figure 2b,c): for NiO^b^/Co/NiO^t^, $H_{C}$ and the $H_{\mathrm{EB}}$ fields are almost two times larger than those of NiO^b^/Co/Au and Au/Co/NiO [36]. This indicates that EB coupling is a sum from both interfaces in NiO^b^/Co/NiO^t^, showing the additive nature of this coupling. Sort et al. [27] reached the same conclusion in their investigations of AFM/FM/AFM systems. In contrast to that work, our study focuses on the additive nature of EB coupling with variable FM thickness. This results in high values of magnetic properties important for applications, e.g., $H_{C,\max}$ = 171 mT at $t_{\mathrm{Co}}$ = 0.83 nm and $H_{\mathrm{EB},\max}$ = 45 mT at $t_{\mathrm{Co}}$ = 0.55 nm for NiO^b^/Co/NiO^t^ system.

We now proceed to confirm the origin of the PMA using values of surface ($K_{S}$) and volume ($K_{V}$) contributions to effective anisotropy ($K_{\mathrm{eff}}$). Anisotropy field values ($H_{K}$) are determined from PMOKE hysteresis loops (Figure 1c,f) for Co thicknesses above the SRT. Then, $K_{\mathrm{eff}}$ is calculated using:

(1) $$K_{\mathrm{eff}}=\frac{-\mu_{0}M_{S}H_{K}}{2}$$

where $\mu_{0}$ is the vacuum permeability and $M_{S}$ is the saturation magnetization of bulk Co. Here, we compare the data with similar Au/Co/Au and Au/Co/NiO systems; therefore, we assume the same saturation magnetization $M_{S}$ = 1.42 × 10^6^ A/m [36]. Linear fits using $K_{\mathrm{eff}}t_{\mathrm{Co}}\;=\;2K_{S}\;+\;K_{V}t_{\mathrm{Co}}$ and $K_{\mathrm{eff}}t_{\mathrm{Co}}$ data (Figure 3) provide the values for $K_{S}$ and $K_{V}$, as summarized in Table 2.

**Figure 5:**
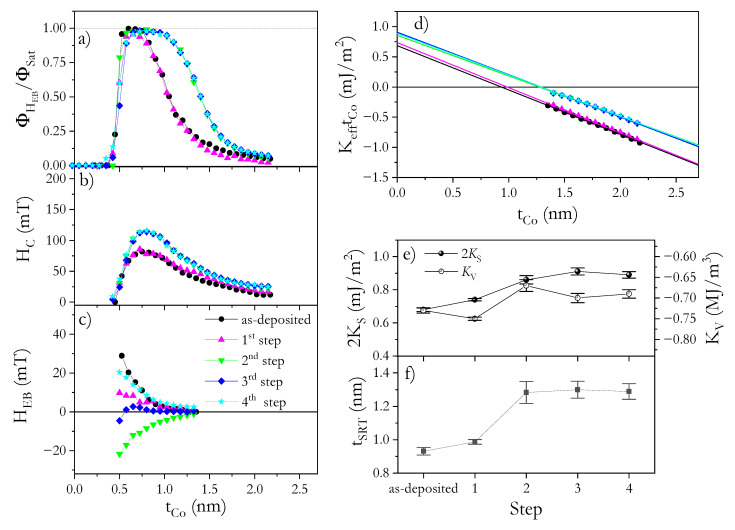
Normalized Kerr signal ($\phi_{H_{\mathrm{EB}}}$/$\phi_{\mathrm{Sat}}$) at *H* = $H_{\mathrm{EB}}$ (**a**), coercivity ($H_{C}$) (**b**) and exchange bias field ($H_{\mathrm{EB}}$) (**c**) as functions of Co layer thickness for NiO^b^/Co/Au. Product of effective magnetic anisotropy and Co thickness ($K_{\mathrm{eff}}t_{\mathrm{Co}}$) as a function of $t_{\mathrm{Co}}$ (**d**) for NiO^b^/Co/Au in the as-deposited state and after different FC steps. Volume and surface anisotropy constants (**e**) and SRT thickness (**f**) for NiO^b^/Co/Au in the as-deposited state and after four different FC steps (1st—350 K, +170 mT; 2nd—450 K, +170 mT; 3rd—350 K, −170 mT; 4th—450 K, −170 mT).

**Figure 6:**
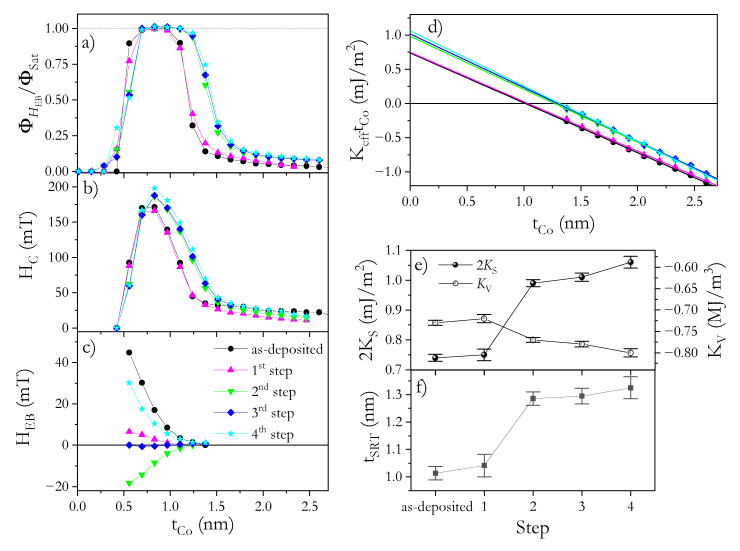
Normalized Kerr signal ($\phi_{H_{\mathrm{EB}}}$/$\phi_{\mathrm{Sat}}$) at *H* = $H_{\mathrm{EB}}$ (**a**), coercivity ($H_{C}$) (**b**) and exchange bias field ($H_{\mathrm{EB}}$) (**c**) as functions of Co layer thickness for NiO^b^/Co/NiO^t^. Product of effective magnetic anisotropy and Co thickness ($K_{\mathrm{eff}}t_{\mathrm{Co}}$) as a function of $t_{\mathrm{Co}}$ (**d**) for NiO^b^/Co/NiO^t^ in the as-deposited state and after different FC steps. Volume and surface anisotropy constants (**e**) and SRT thickness (**f**) for NiO^b^/Co/NiO^t^ in the as-deposited state and after four different FC steps (1st—350 K, +170 mT; 2nd—450 K, +170 mT; 3rd—350 K, −170 mT; 4th—450 K, −170 mT).

The data in Table 2 show that the surface contribution (2$K_{S}$) to the effective anisotropy on both of our systems is similar to those on Au/Co/Au [2,36,43] and on Pt/Co/Pt [2,3]. In comparison with HM/Co/Oxide systems, our values are two times smaller [44,45]; however, after low-temperature annealing during the FC procedure, these values increase significantly (see Figure 5e and Figure 6e), similar to what was found in Ref. [44]. Note that a direct comparison of individual surface contributions to PMA from the Co/oxide interfaces is quite difficult because typical studies of oxide interface effects on PMA are performed for HM/Co/oxide systems, where the FM layer is adjacent to HM (e.g., Au, Pt, Pd), providing high interface anisotropy [2,3]. To get information about $K_{S}$ for the Co/oxide interface, the contribution from HM/Co must be subtracted from 2$K_{S}$ and it is usually determined from symmetrical HM/Co/HM systems with the assumption that both HM/Co and Co/HM interfaces are identical and contribute equally to surface anisotropy. Hence, the determination of these values is often approximated under this assumption. Nevertheless, the NiO^b^/Co/NiO^t^ data clearly show that the AFO/FM (FM/AFO) interface is a source of PMA, with $K_{S}$ of a similar order of magnitude to those of HM/FM.

If the NiO^t^ gives a higher $K_{S}$ than Au [36], the 2$K_{S}$ value should be higher for NiO^b^/Co/NiO^t^ than for NiO^b^/Co/Au. Indeed, this is the case for our studies (Table 2). A larger 2$K_{S}$ also explains the shift of the SRT to larger $t_{\mathrm{Co}}$ (Table 2). Note that, for the NiO^b^/Co/Au and NiO^b^/Co/NiO^t^ systems, the *K*_V_ values are identical and equal to the sum of shape anisotropy for Co thin films (−1/2$\mu_{0}M_{S}$^2^ = −1.27 MJ/m^3^) and magnetocrystalline anisotropy for the hexagonal structure of Co (0.53 MJ/m^3^) [2]. This indicates that magnetocrystalline anisotropy enhances PMA when Co is deposited on a NiO layer; however, we cannot exclude additional contribution to the $K_{V}$, e.g., from magnetoelastic anisotropy. It should be emphasized that to date, the PMA has been investigated mainly in HM/FM/oxide systems, where a strong Co/HM surface anisotropy also helps to stabilize the PMA. Here, we demonstrate that HM is not necessary to stabilize PMA at RT, which offers a new type of multilayer system with strong PMA.

### 3.2. Magnetic Properties of NiO^b^/Co/Au and NiO^b^/Co/NiO^t^ Systems after Different FC Steps

To tune the EB coupling, the NiO^b^/Co/Au and NiO^b^/Co/NiO^t^ systems underwent the four FC steps described above (see Table 1 in the Experiment section). After the first FC step ($T_{\mathrm{FC}}$ = 350 K in $H_{\mathrm{FC}}$ = +170 mT), the $H_{\mathrm{EB}}$ reduces significantly (Figure 5c and Figure 6c) but coercivity (Figure 5b and Figure 6b) and effective anisotropy (Figure 5d and Figure 6d) maintain their high values for both systems. Note that the direction of $H_{\mathrm{FC}}$ is opposite to the direction of $H_{\mathrm{dep}}$. One would expect a flip in the EB coupling direction for FC processes starting at $T_{\mathrm{FC}}$ higher than the Néel (blocking) temperature $T_{N}$($T_{B}$). This is not observed in our case, although $H_{\mathrm{EB}}$ experiences a significant decrease. In the analysis of polycrystalline samples (Figure 4), the AFM layer is usually treated as a set of magnetically noninteracting grains with a size distribution that spreads the $T_{B}$ [46,47]. Therefore, grains with $T_{B}$ < $T_{\mathrm{FC}}$ lose their AFM pinning strength and, during the FC procedure, the pinning direction may reverse from the initial direction set by $H_{\mathrm{dep}}$ to the direction parallel to $H_{\mathrm{FC}}$. For NiO^b^/Co/Au, this means that part of the grain is coupled along $H_{\mathrm{FC}}$ and part along $H_{\mathrm{dep}}$ (i.e., in opposite directions), and as a result, the effective $H_{\mathrm{EB}}$ is strongly reduced.

In the case of NiO^b^/Co/NiO^t^, we need to consider EB effect contributions from two interfaces simultaneously (NiO/Co and Co/NiO^t^), and the fact that EB couplings at each interface are too weak to introduce rotation of the Co spin across the film thickness if the EB coupling direction at both interfaces is opposite. This is due to the small thickness and higher exchange and anisotropy energies than EB coupling energy. Since after the first step of FC we found that the $H_{\mathrm{EB}}$ for NiO^b^/Co/Au is slightly larger than for NiO^b^/Co/NiO^t^, many more grains at the Co/NiO^t^ interface are coupled along $H_{\mathrm{FC}}$ than at the NiO^b^/Co interface. This means that the grains of NiO^b^ show a lower blocking temperature than NiO^t^, which can be correlated with CoO at the Co/NiO^t^ interface, which reduces the ordering (blocking) temperature of NiO [48]. It should be emphasized that, at this low annealing temperature (350 K), only $H_{\mathrm{EB}}$ changes significantly, showing that annealing below 350 K can be used to tune this parameter without altering magnetic anisotropy.

To couple even more grains, a second FC step was applied starting from a higher temperature ($T_{\mathrm{FC}}$ = 450 K) and with the same value and direction of $H_{\mathrm{FC}}$ (+170 mT). In this step, we expected that the temperature was high enough to align the EB coupling of many more grains with $H_{\mathrm{FC}}$ for both systems. Indeed, $H_{\mathrm{EB}}$ became highly negative (Figure 5c and Figure 6c), indicating a strongly effective EB. However, the magnitude of $H_{\mathrm{EB}}$ was smaller, especially for NiO^b^/Co/NiO^t^, than for the as-deposited state, which could have been caused by interface modification during annealing. This statement also supports an additional observation: the SRT occurs at a larger $t_{\mathrm{Co}}$ (Figure 5d,f and Figure 6d,f), and the corresponding increase in PMA (Figure 5d and Figure 6d) correlates with an increase in $H_{C}$ (Figure 5b and Figure 6b). Therefore, at this step, irreversible changes in the microstructure take place, which are stable for further annealing up to 450 K (see Figure 5 and Figure 6 for 3rd and 4th steps). Since the second FC process did not result in the shift of $t_{\mathrm{Co}}$ where PMA starts to appear (Figure 5a and Figure 6b), and we do not detect any additional shift of $\phi_{\mathrm{Sat}}$($t_{\mathrm{Co}}$) dependence, we assume that there is no further oxidation of the Co layer and therefore the anisotropy changes are not related to reductions in $t_{\mathrm{Co}}$. Note that for both systems, $K_{V}$ almost does not change ($K_{V}$ = 0.67 MJ/m^3^ and $K_{V}$ = 0.77 MJ/m^3^ for NiO^b^/Co/Au and NiO^b^/Co/NiO^t^ systems, respectively) (Figure 5e and Figure 6e); therefore, additional oxidation of Co layer can be excluded after the FC process. Thus, the increase in PMA is attributed to interface morphology modifications because 2$K_{S}$ increases to 0.86 mJ/m^2^ for NiO^b^/Co/Au (Figure 5e) and to 0.99 mJ/m^2^ NiO^b^/Co/NiO^t^ (Figure 6e). A similar increase in $K_{S}$ was shown for HM/Co/oxide systems after annealing, which was attributed to homogeneous oxidation along the interface and to interface smoothening [44]. Considering that this type of interface modification might be a source of smaller $H_{\mathrm{EB}}$ [49] and that 2$K_{S}$ increase less for the NiO^b^/Co/Au system than for NiO^b^/Co/NiO^t^, we expect that the changes on the Co/NiO^t^ interface are greater than those on the NiO^b^/Co interface, which may be a source of the lower $H_{\mathrm{EB}}$ for NiO^b^/Co/NiO^t^.

The last two FC steps described in Table 1 help us to understand the effects of PMA enhancement on EB coupling. After these steps, for both systems, neither effective magnetic anisotropy (Figure 5d–f and Figure 6d–f) ($K_{S}$ and $K_{V}$, and $t_{\mathrm{SRT}}$) nor $H_{C}$ show significant changes. This means that strong interface modifications caused by annealing at *T* ≤ 450 K have ceased, and the reversible $H_{\mathrm{EB}}$ changes in the 3rd and 4th step can be repeated without altering other magnetic properties.

Note that in the 4th step, the $H_{\mathrm{FC}}$ is aligned in the same direction as $H_{\mathrm{dep}}$, therefore we should expect that pinning directions from all NiO^b^ and NiO^t^ grains are aligned in the same direction giving a high $H_{\mathrm{EB}}$ effect. Indeed, these values are high; however, $H_{\mathrm{EB}}$ is smaller than that observed for the as-deposited state, which we attribute to a smaller contribution to effective EB field from Co/NiO^t^ appearing after the 2nd step of the FC process. Nevertheless, $H_{\mathrm{EB}}$ and $H_{C}$ for NiO^b^/Co/NiO^t^ (Figure 6b,c) are still much higher than for NiO^b^/Co/Au (Figure 5b,c) showing additive nature of EB coupling. All that shows that a carefully chosen AFM–FM system offers simultaneous support for a strong PMA and high $H_{\mathrm{EB}}$, which can be tuned over a wide range by the proper selection of an FC procedure.

## 4. Conclusions

In summary, the EB coupling in NiO/Co and Co/NiO interfaces has been investigated in terms of perpendicular magnetic anisotropy and EB field. Using NiO/Co/Au and NiO/Co/NiO systems, we have shown that the CoNiO interface induces strong surface contribution to the effective magnetic anisotropy, favoring out-of-plane magnetization of Co layer. We also demonstrate that strong perpendicular magnetic anisotropy can be achieved by using only AFO-FM interfaces, where the EB field can be modified over a wide range by proper selection of field cooling conditions. The presence of two interfaces, NiO/Co and Co/NiO, in a NiO/Co/NiO system allows us to reach high $H_{C}$ and $H_{\mathrm{EB}}$ because each interface simultaneously supports EB coupling and PMA. These results establish that a new multilayer system based on antiferromagnetic oxides offers strong PMA and the ability to tune $H_{\mathrm{EB}}$ and $H_{C}$ in a wide range, which are important qualities for spintronic applications.

## Figures and Tables

**Table 1:** Combinations of heating temperatures and signs of $H_{\mathrm{FC}}$ for each field cooling (FC) step.

Steps	$T_{\mathrm{FC}}$ (K)	$H_{\mathrm{FC}}$ (mT)
1st	350	+170
2nd	450	+170
3rd	350	−170
4th	450	−170

**Table 2:** Volume and surface anisotropy constants ($K_{V}$, $K_{S}$) and $t_{\mathrm{Co}}$ at SRT thickness ($t_{\mathrm{SRT}}$) for NiO^b^/Co/Au and NiO^b^/Co/NiO^t^ in the as-deposited state.

System	$K_{V}$ (MJ/m^3^)	$2K_{S}$ (mJ/m^2^)	$t_{\mathrm{SRT}}$ (nm)
NiO^b^/Co/Au	−0.73 ± 0.01	0.68 ± 0.01	0.93 ± 0.01
NiO^b^/Co/ NiO^t^	−0.73 ± 0.01	0.74 ± 0.01	1.01 ± 0.01
Au/Co/Au [36]	−0.58	0.65	1.12
Au/Co/NiO/Au [36]	−1.06	1.4	1.32

## Data Availability

The data presented in this study are available on request from the corresponding author.
